# Evaluation of Change in the Facial Profile and Aesthetics in Relation to Incisor Position in Both Maxillary and Mandibular Arches

**DOI:** 10.7759/cureus.34403

**Published:** 2023-01-30

**Authors:** Abhimanyu V Singh, Avinash Mahamuni, Jyoti S Gaharwar, Rajlaxmi Rai, Kavita Yadav, C Sirishkusum

**Affiliations:** 1 Department of Orthodontics and Dentofacial Orthopedics, Babu Banarasi Das Dental College, Lucknow, IND; 2 Department of Orthodontics and Dentofacial Orthopedics, Late Shri Yashwantrao Chavan Dental College, Ahmednagar, IND; 3 Department of Orthodontics, Amaltas Institute of Medical Sciences, Dewas, IND; 4 Department of Orthodontics, Purvanchal Institute of Dental Sciences, Gorakhpur, IND; 5 Department of Orthodontics, Employees' State Insurance Corporation, Gurugram, IND

**Keywords:** facial profile, facial harmony, orthodontic, soft tissue, incisor, aesthetics

## Abstract

Background

The orthodontist is often confronted with the need to predict soft tissue profile changes that may result from the orthodontic treatment. The problem arises because the contribution of many of the factors influencing the soft tissue profile still needs to be fully understood. The complexity of the problem is increased in growing patients in whom the post-treatment soft tissue profile is the result of both growth and orthodontic treatment. A primary motivation for seeking orthodontic treatment is a desire to improve dental and facial aesthetics. To achieve balance in the facial profile treated orthodontically, it is essential to identify the underlying skeletal hard tissue and soft tissue parameters. The present study evaluated the changes in facial profile and aesthetics in relation to incisor position.

Materials and methods

Samples for this study consisted of pre-treatment lateral cephalograms of the Indian population (n = 450) having different incisor relationships. Subjects aged between 18 and 30 years were included. Angular and linear measurements were taken to analyse the incisor relationship with soft tissue parameters.

Results

The majority (61.2%) of subjects belonged to the age group of 18-30 years. The overall female-to-male ratio in the study was 7:3. The parameter U1 to L1 was abnormal in 86.8% of subjects. Similarly, the parameters S-line upper lip (UL), S-line lower lip (LL), E-line UL, and E-line LL were found abnormal in 93.9%, 86.8%, 82.6%, and 70.1% of subjects, respectively. A significant agreement was found between U1 to L1 and E-line UL and U1 to L1 and E-line LL.

Conclusions

The present study concludes that facial aesthetics combines soft and hard tissue corrections, not just based on occlusal relationships but also considering facial harmony. Thus, the incisor relationship is an important asset and strongly correlates with other soft tissue and hard tissue parameters that improve facial aesthetics for the individual undergoing orthodontic treatment.

## Introduction

The orthodontist is often confronted with the need to predict soft tissue profile changes that may result from the orthodontic treatment. The problem arises because the contribution of many of the factors influencing the soft tissue profile still needs to be fully understood [1]. The complexity of the problem is increased in growing patients in whom the post-treatment soft tissue profile is the result of both growth and orthodontic treatment. A primary motivation for seeking orthodontic treatment is a desire to improve dental and facial aesthetics [2,3]. In planning correction for a patient with a convex profile, the clinician must consider the soft tissue changes that may occur with the correction of the anteroposterior position of the maxillary incisors [4]. The incisor classification of malocclusion has enjoyed wide acceptance since it was introduced by A.C. Williams and has now superseded Angle's classification in the United Kingdom [1]. The problem was well summarised by Kazutaka Kasai, who notes that mechanotherapy to obtain a functional occlusion may compromise facial aesthetics in some patients [5]. The soft tissue response is a proportion of incisors and results in inherent spatial, functional, and structural features. To obtain the balance in the facial profile treated orthodontically, it is essential to identify the underlying skeletal hard tissue and soft tissue parameters [6]. The present study evaluated the changes in facial profile and aesthetics in relation to incisor position.

## Materials and methods

This retrospective cohort study was performed in the Department of Orthodontics and Dentofacial Orthopaedics and the Department of Oral Medicine and Radiology, Saraswati Dental College, Lucknow. The Institutional Human Ethical Committee (IHEC) at Saraswati Dental College, Lucknow approved the study (SDC/IHEC/2016/MDS/16). Samples for this study consisted of pre-treatment lateral cephalograms of the North Indian population (n = 450) having different incisor relationships. Subjects were between 18 and 30 years old, and all subjects were born in and were residents of Uttar Pradesh. Each subject met the following inclusion criteria: radiographs selected according to the British system of incisor classification (Class I, Class II, and Class III relationship), aged between 18 and 30 years, no history of previous orthodontic treatment, no history of trauma, and no other congenital anomaly.

Standardisation of lateral cephalogram radiograph

While recording the lateral cephalograms, patients stood with the Frankfort horizontal plane parallel to the floor and the teeth in centric occlusion. The heads of the patients were erect. All cephalograms were recorded with the same exposure parameters (KvP: 80; mA: 10; exposure time: 0.5 seconds) with 100% magnification and with the same machine (Kodak 8000C Digital and Panoramic System Cephalometer, Carestream Health, Rochester, NY). The cephalogram was exposed at the end-expiration phase of the respiration. The lateral cephalogram was given initials and was standardised using a cross of 2 x 2 inches with a serial number on every cephalogram using lead alphabets and digits. The X-rays were printed using Fujifilm Medical Dry Imaging film (8 x 10 inches in size) and the Fujifilm Dry PIX Plus printer (Fujifilm Healthcare, Lexington, MA). Angular and linear parameters were measured as described below.

Angular Measurements

U1 to L1 (interincisal angle): The interincisal angle is established by passing a line through the incisal edge and the apex of the root of the maxillary and mandibular central incisors.

Z-angle line: The Z-angle line is established by drawing a line tangent to soft tissue pogonion and to the most anterior point of either the lower or upper lip, whichever is more protrusive. The intersection of the Frankfort horizontal plane and the profile line forms the angle.

H-line angle: The H-line is a tangent to menton and labial superioris. The H-line angle is formed between this line and the soft tissue nasion and pogonion.

Nasolabial angle: The nasolabial angle is formed by drawing a line tangent to the base of the nose and a line tangent to the upper lip.

Linear Measurements

S-line: The Steiner line or S-line is drawn from soft tissue pogonion to the midpoint of the S-shaped curve between subnasale and pronasale.

E-line: The E-line (aesthetic line of Ricketts) is drawn from pronasale to soft tissue pogonion. Various planes are marked in lateral cephalograms.

F-H plane: The plane is formed by drawing a straight line through the bony margins of the orbit to the upper margins of the external auditory meatus. This plane is used to describe malocclusions in a vertical plane.

Reliability analysis

For reliability analysis, 10 randomly selected two observers measured cephalograms. Each observer did two readings. A single observer's average of two readings was taken as the representative measurement. The representative measurements of two observers were compared using Dahlberg's error analysis. Dahlberg's error is <0.5 mm for all the parameters, which is well below the acceptability criteria of 0.5 mm. Hence, the observations were considered normal (Table 1).

**Table 1:** Dahlberg's error analysis

Parameter	Dahlberg's error
U1 to L1	0.02
S-line (upper lip)	0.01
S-line (lower lip)	0.02
E-line (upper lip)	0.05
E-line (lower lip)	0.01

Statistical data

Data were summarised as mean ± standard deviation (SD). Independent Student’s t-test compared groups. Categorical (discrete) groups were compared by the chi-square (χ2) test. Pearson correlation analysis was done to assess the association between the variables. A two-tailed (α = 2) p < 0.05 was considered statistically significant.

## Results

The majority (61.2%) of the subjects belonged to the age group of 18-30 years. The mean age of the subjects was 20.61 ± 2.54 years. Among 425 subjects, 299 (70.4%) were females, and the rest (126, 29.6%) were males. So the study's overall female-to-male ratio was 7:3.

The parameter U1 to L1 was abnormal in 86.8% of subjects, and it was normal among the rest 13.2% of subjects. Similarly, the parameters S-line upper lip (UL), S-line lower lip (LL), E-line UL, and E-line LL were found abnormal in 93.9%, 86.8%, 82.6%, and 70.1% of subjects, respectively (Table 2).

**Table 2:** Status of hard tissue facial parameters UL: upper lip; LL: lower lip.

Parameters	Normal		Abnormal	
	No.	%	No.	%
U1 to L1	56	13.2	369	86.8
S-line UL	26	6.1	399	93.9
S-line LL	56	13.2	369	86.8
E-line UL	74	17.4	351	82.6
E-line LL	127	29.9	298	70.1

The parameters U1 to L1 and S-line UL were abnormal in 81.2% of subjects and normal in 0.5%. No significant agreement (k = 0.038, p = 0.394) was found between the two parameters (Table 3).

**Table 3:** Agreement between U1 to L1 parameter and S-line UL UL: upper lip.

Parameter			S-line UL		Total	Kappa	P-value
			Normal	Abnormal			
U1 to L1	Normal	No.	2	54	56	-0.038	0.394
		%	.5%	12.7%	13.2%		
	Abnormal	No.	24	345	369		
		%	5.6%	81.2%	86.8%		
Total		No.	26	399	425		
		%	6.1%	93.9%	100.0%		

The parameters U1 to L1 and S-line LL were abnormal in 77.9% of subjects and normal in 4.2%. In addition, a significant agreement (k = 0.218, p < 0.001) was found between the two parameters (Table 4).

**Table 4:** Agreement between U1 to L1 parameter and S-line LL LL: lower lip.

Parameter			S-line LL		Total	Kappa	P-value
			Normal	Abnormal			
U1 to L1	Normal	No.	18	38	56	0.218	<0.001
		%	4.2%	8.9%	13.2%		
	Abnormal	No.	38	331	369		
		%	8.9%	77.9%	86.8%		
Total		No.	56	369	425		
		%	13.2%	86.8%	100.0%		

The parameters U1 to L1 and E-line UL were abnormal in 75.5% of subjects and normal in 6.1%. In addition, a significant agreement (k = 0.294, p < 0.001) was found between the two parameters (Table 5).

**Table 5:** Agreement between U1 to L1 parameter and E-line UL UL: upper lip.

Parameter			E-line UL		Total	Kappa	P-value
			Normal	Abnormal			
U1 to L1	Normal	No.	26	30	56	0.294	<0.001
		%	6.1%	7.1%	13.2%		
	Abnormal	No.	48	321	369		
		%	11.3%	75.5%	86.8%		
Total		No.	74	351	425		
		%	17.4%	82.6%	100.0%		

The two parameters U1 to L1 and E-line LL were found abnormal in 64.2% of subjects and normal in 7.3% of subjects. A significant agreement (k = 0.191, p < 0.001) was found between the two parameters (Table 6).

**Table 6:** Agreement between U1 to L1 parameter and E-line LL LL: lower lip.

Parameter			E-line LL		Total	Kappa	P-value
			Normal	Abnormal			
U1 to L1	Normal	No.	31	25	56	0.191	<0.001
		%	7.3%	5.9%	13.2%		
	Abnormal	No.	96	273	369		
		%	22.6%	64.2%	86.8%		
Total		No.	127	298	425		
		%	29.9%	70.1%	100.0%		

The two parameters S-line UL and S-line LL were abnormal in 80.7% of subjects and normal in 0.0% of subjects. In addition, a significant disagreement (k = -0.091, p = 0.040) was found between the two parameters (Table 7).

**Table 7:** Agreement between S-line UL and S-line LL UL: upper lip; LL: lower lip.

Parameter			S-line LL		Total	Kappa	P-value
			Normal	Abnormal			
S-line UL	Normal	No.	0	26	26	-0.091	0.040
		%	0.0%	6.1%	6.1%		
	Abnormal	No.	56	343	399		
		%	13.2%	80.7%	93.9%		
Total		No.	56	369	425		
		%	13.2%	86.8%	100.0%		

The two parameters S-line UL and E-line UL were found abnormal in 77.6% of subjects and normal in 1.2% of subjects. No significant agreement (k = 0.01, p = 0.801) was found between the two parameters (Table 8).

**Table 8:** Agreement between S-line UL parameter and E-line UL UL: upper lip.

Parameter			E-line UL		Total	Kappa	P-value
			Normal	Abnormal			
S-line UL	Normal	No.	5	21	26	0.01	0.801
		%	1.2%	4.9%	6.1%		
	Abnormal	No.	69	330	399		
		%	16.2%	77.6%	93.9%		
Total		No.	74	351	425		
		%	17.4%	82.6%	100.0%		

## Discussion

The present study is correlative. The present study was conducted in the Department of Orthodontics and Dentofacial Orthopaedics, Saraswati Dental College, Lucknow. The sample size for this study consisted of subjects from the Indian population (n = 425) aged between 18 and 30 years. The study included late pubertal and early subjects, and all subjects were born in and were residents of Uttar Pradesh. The entire sample was divided based on gender (male = 126 and female = 299). The pretreatment lateral cephalogram of untreated patients was taken from the Department of Orthodontics and Dentofacial Orthopaedics, Saraswati Dental College.

The subjects included in the present study were of the age group of 18-30 years. This finding was supported by Hasund et al., who conducted a study to evaluate lower incisors that showed a positive correlation with soft tissue parameters in subjects aged 18 years [7]. Similarly, Lamberton et al. conducted a study to determine the nature and occurrence of bimaxillary protrusion in subjects aged 22 years [8]. In the same way, El Kaki et al. conducted a study to assess the soft tissue measurements of Moroccan adolescents with a balanced facial profile on subjects aged between 18 and 20 years, and Patel et al. also conducted a study to evaluate different soft tissue structures for effective orthodontic treatment on subjects aged 18-25 years and found a significant association between upper and lower lip to S-line and E-line [9,10].

In the present study, the frequency of female and male subjects was 70% and 30%, which was an uneven distribution. These findings are in accordance with those observed by Lamberton et al., who conducted a study to determine the nature and occurrence of bimaxillary protrusion in 29 males and 46 females, which was also an uneven distribution [8]. On the contrary, Subtelny conducted a study in which an equal number of male and female subjects with normal skeletal profiles were included [11]. Similarly, Chaconas et al. conducted a study to evaluate the importance of facial profiles in orthodontic therapy on an equal number of male and female subjects [12].

In the present study, according to the status of hard tissue, facial parameters from U1 to L1 were found to be abnormal in 86.8% of subjects and normal in 13.2% of subjects. These findings are consistent with those observed by Moore, who also found that various changes can be made in the growth phase in the hard tissue parameters of the patient [13].

Furthermore, similar findings were reported by Fishman, who also found that facial imbalance occurred due to both maxillary and mandibular hard tissue parameters at different patient chronological and skeletal ages [14].

In the current study, the status of soft tissue facial parameters was found to be abnormal. The findings were in correlation with Roos, who conducted a study to contribute information on soft tissue profile and found that the soft tissue profile does not, in all respects, directly reflect changes in the underlying skeletal profile during orthodontic treatment [15]. Similarly, Merrifield also found that young female patients after orthodontic treatment have a better chin-lip relationship than young males [16]. In the same way, Prahl-Andersen et al. conducted a study on the growth of the lips, nose, and chin in mixed dentition and found an increase in the thickness of the soft tissue at pogonion. In addition, growth in girls decreases after nine years, whereas boys show a growth spurt at 14 years [17].

In the present study, the agreement between U1 to L1 and S-line upper lip was insignificant (p = 0.394). These findings were in accordance with those observed by Roos, who had conducted a study to contribute to the existing pool of information on the soft tissue profile of 30 patients with class II division I malocclusion [15]. They found that the soft tissue profile does not directly reflect changes in the underlying skeletal profile during orthodontic treatment.

On the contrary, Namratha et al. conducted a study to compare and evaluate the upper lip length parameter on 80 subjects of the South Indian population and found that the relationship of the upper lip to the maxillary incisor of the mouth is significant [18]. Likewise, Prahl-Andersen et al. conducted a study on the growth of the lips, nose, and chin in mixed dentition and found an increase in the thickness of the soft tissue at pogonion [17].

The parameters U1 to L1 and S-line lower lip in the present study showed a significant association (p < 0.001). The findings were supported by Kasai, who conducted a study to investigate soft tissue adaptability to hard tissue in a static state on 297 Japanese women and found that in the static state, the vertical dimension of lower facial height and the position of the lower incisors were associated with the thickness of the upper lips and pogonion [5]. The results indicated that the soft tissue strongly reflected the changes in the hard tissue. In the same way, Singh conducted a study to evaluate changes in the contour of the lower lip immediately after orthodontic treatment on 50 subjects and found an increase in the thickness of the lower lip after treatment [19]. On the contrary, Rains et al. conducted a study to determine the response of the lower lip to maxillary and mandibular incisor movement and found that lower incisor movement did not correlate with the change in the lower lip [3].

The current study found a significant association between U1 to L1 and E-line upper lip (p < 0.001). These findings were in accordance with the finding observed by Fishman, who conducted a study to investigate the comparison that exists between the chronological and skeletal ages within a population of 382 subjects and found that facial imbalances occurred due to both maxillary and mandibular hard and soft tissue parameters at different chronological and skeletal age of the patient [14]. Similarly, Forsberg et al. conducted a study to know the importance of specific growth changes that occurred in the relationship between the lips and E-line and showed that there were some sex differences, which should be borne in mind [20]. Likewise, Oliver conducted a study to investigate the influence of maxillary lip thickness on the relationship between dental and integumental tissue changes on 40 Caucasian subjects with class II division I malocclusion and found the correlation between osseous and soft tissue changes was significant [21].

A significant agreement was found between the two parameters U1 to L1 and E-line lower lip (p < 0.001). On the contrary, Yashutomi et al. conducted a study to evaluate the upper and lower lip changes after orthodontic treatment of bimaxillary protrusion in Japanese adults and found no significant differences in the mean variables between the subjects [22]. Similarly, Hayashida et al. conducted a study to determine the effects of retraction of anterior teeth and the initial soft tissue profile variables on upper and lower lip changes and found a significant correlation between the upper and lower lip and retraction of anterior teeth [23].

The two parameters, S-line upper lip and E-line upper lip, showed no significant association (p = 0.801). On the contrary, Saad et al. conducted a study to find out the anteroposterior position of lips in photographs using E-line and S-line in patients with orthognathic profiles and to establish a correlation between lip prominence judged by E-line and S-line on 90 subjects and found a statistically significant correlation between upper lip to E-line and upper lip to S-line [24].

The present study found a significant agreement between the S-line upper lip and E-line lower lip (p < 0.001). This finding was in correlation with Verma et al., who conducted a study to analyse the soft tissue changes between different treatment groups equally susceptible to both treatment options and compare the changes taking place in the soft tissue variables from one group to another group using cephalometric analysis and found that significant differences regarding upper lip to S-line, lower lip protrusions, and upper lip thickness occurred after orthodontic treatment [25].

In the current study, a significant association was found between the two parameters S-line lower lip and E-line upper lip (p < 0.001). This finding coincided with the findings of Bokari et al., who conducted a study to find out the anteroposterior position of lips on cephalogram by S-line in patients with orthognathic profile and to establish a correlation between determinants of lip prominence on 65 subjects and found that a statistically significant correlation existed between lower lip prominence as assessed by S-line and upper lip prominence using E-line [26].

The parameters between the S-line lower lip and E-line lower lip in the present study showed a significant agreement (p < 0.001). This finding was in line with Forsberg et al., who conducted a study on 120 healthy Swedish boys and girls with normal occlusion and found the growth changes and soft tissue of individuals receiving orthodontic treatment. They found that growth changes that occur in the relationship between the lips and E-line and S-line show that there is a significant association between these two parameters [20].

On the contrary, Amirabadi et al. conducted a study to evaluate soft tissue changes after upper first premolar extraction in 20 class II division I cases with average vertical height and found no significant difference in the upper and lower lip relationship to E-line and S-line [27].

The present study found a significant agreement between the E-line upper lip and E-line lower lip (p < 0.001). This finding was supported by Chalipa et al., who conducted a study to determine cephalometric changes of facial soft tissue after combined treatment in patients with class III discrepancy on 25 subjects and found that upper and lower lip to E-line distance and angle of convexity changed significantly [28].

The vertical dimension of incisors is mostly determined by lip contour; at rest, the lower edge of the upper incisors should touch the upper vermillion of the lower lip [29,30]. When smiling, most orthodontists and dentists prefer that the elevation of the lip stops at the gingival margins of the maxillary incisors; some amount of gingival display is certainly acceptable and, in many cases, is even aesthetic and results in a youthful appearance [31-33]. The absence of alignment between the lower lip and the edge of the maxillary incisors detracts from the beauty of the smile in both the frontal and lateral views [31]. Moreover, the buccolingual inclination of the maxillary incisors has a major effect on profile smile attractiveness [32,33]. To quantify innate feelings about the impact of incisor inclination on smile aesthetics, an anchored scale (visual analogue scale) was used. This method has been endorsed by many investigators for use in attractiveness ratings because of its simplicity and ease of use and it avoids the bias towards preferred values that are found with numeric or interval scales and allows a better examination of the amount and significance of differences [34,35].

The limitation of this study is that it has been performed on a two-dimensional cephalogram; hence, the measurement error is present. Also, the study is performed on the population of Uttar Pradesh, India. Hence, the results are only constrained to a specific region and may vary due to variations in ethnic origin.

## Conclusions

The present study concludes that facial aesthetics combines soft and hard tissue correction, not just based on occlusal relationships but also considering facial harmony. Thus, the incisor relationship is an important asset and has strong correlations with other soft tissue and hard tissue parameters that provide improved facial aesthetics to the individual undergoing orthodontic treatment.

## References

[1] Williams AC, Stephens CD (1992). A modification to the incisor classification of malocclusion. Br J Orthod.

[2] Al-Maqtari RA, Kadir RA, Awang H, Zam Zam NM (2012). Assessment of malocclusion among Yemeni adolescents using canine and incisor classifications. J Adv Med Res.

[3] Rains MD, Nanda R (1982). Soft-tissue changes associated with maxillary incisor retraction. Am J Orthod.

[4] Waldman BH (1982). Change in lip contour with maxillary incisor retraction. Angle Orthod.

[5] Kasai K (1998). Soft tissue adaptability to hard tissues in facial profiles. Am J Orthod Dentofacial Orthop.

[6] Islam M, Uraibi AH, Al Azzawi A, Alam MK, Yusof A (2019). Sagittal discrepancies of the jaw in a Bangladeshi cohort: three-dimensional computed tomography analysis. J Int Med Res.

[7] Hasund A, Ulstein G (1970). The position of the incisors in relation to the lines NA and NB in different facial types. Am J Orthod.

[8] Lamberton CM, Reichart PA, Triratananimit P (1980). Bimaxillary protrusion as a pathologic problem in the Thai. Am J Orthod.

[9] El Kaki S, Ousehal L (2018). Soft tissue cephalometric standards for a Moroccan teenage population. Int J Dentistry Oral Sci.

[10] Patel B, Goyal K (2017). Determination of soft tissue cephalometric norms of Rajasthan population using Holdway analysis. J Den Med Sci.

[11] Subtelny JD (1959). A longitudinal study of soft tissue facial structures and their profile characteristics, defined in relation to underlying skeletal structures. Am J Orthod.

[12] Chaconas SJ, Bartroff JD (1975). Prediction of normal soft tissue facial changes. Angle Orthod.

[13] Moore AW (1959). Observations on facial growth and its clinical significance. Am J Orthod.

[14] Fishman LS (1979). Chronological versus skeletal age, an evaluation of craniofacial growth. Angle Orthod.

[15] Roos N (1977). Soft-tissue profile changes in class II treatment. Am J Orthod.

[16] Merrifield LL (1966). The profile line as an aid in critically evaluating facial esthetics. Am J Orthod.

[17] Prahl-Andersen B, Ligthelm-Bakker AS, Wattel E, Nanda R (1995). Adolescent growth changes in soft tissue profile. Am J Orthod Dentofacial Orthop.

[18] Namratha M, Dhanraj M, Jain AR (2017). Comparative evaluation of upper lip length and the commissural height in Chennai population. J Adv Pharm Edu Res.

[19] Singh RN (1990). Changes in the soft tissue chin after orthodontic treatment. Am J Orthod Dentofacial Orthop.

[20] Forsberg CM, Odenrick L (1979). Changes in the relationship between the lips and the aesthetic line from eight years of age to adulthood. Eur J Orthod.

[21] Oliver BM (1982). The influence of lip thickness and strain on upper lip response to incisor retraction. Am J Orthod.

[22] Yashutomi H, Ioi K, Nakata S, Nakasima A, Counts AL (2006). Effects of retraction of anterior teeth on horizontal and vertical lip positions in Japanese adults with the bimaxillary dentoalveolar protrusion. Orthod Waves.

[23] Hayashida H, Ioi H, Nakata S, Takahashi I, Counts AL (2011). Effects of retraction of anterior teeth and initial soft tissue variables on lip changes in Japanese adults. Eur J Orthod.

[24] Asad S, Kazmi F, Mumtaz M, Malik A, Raz Baig R (2011). Assessment of antero-posterior position of lips: E-line - S-line. Pak Oral Dent J.

[25] Verma SL, Sharma VP, Tandon P, Singh GP, Sachan K (2013). Comparison of esthetic outcome after extraction or non-extraction orthodontic treatment in class II division 1 malocclusion patients. Contemp Clin Dent.

[26] Bokari F, Amin F, Asad S (2013). Cephalometric assessment of lips in skeletal class II patients by Steiner's line. Ann King Edw Med Univ.

[27] Amirabadi GE, Mirzaie M, Kushki SM, Olyaee P (2014). Cephalometric evaluation of soft tissue changes after extraction of upper first premolars in class ΙΙ div 1 patients. J Clin Exp Dent.

[28] Chalipa J, Roochi MM, Mortazavi M (2018). Cephalometric evaluation of facial soft tissue changes after orthodontics and bimaxillary orthognathic surgery treatment in patients with skeletal class III discrepancy. Iran J Orthod.

[29] Frindel F (2001). For a better place of the smile (part one). Rev Orthop Dento Faciale.

[30] Zachrisson BU (1998). Esthetic factors involved in anterior tooth display and the smile: vertical dimension. J Clin Orthod.

[31] Sarver DM (2001). The importance of incisor positioning in the esthetic smile: the smile arc. Am J Orthod Dentofacial Orthop.

[32] Sarver DM, Ackerman MB (2003). Dynamic smile visualization and quantification: part 2. Smile analysis and treatment strategies. Am J Orthod Dentofacial Orthop.

[33] Sarver DM, Proffit WR (2005). Special considerations in diagnosis and treatment planning. Orthodontics: Current Principles and Techniques.

[34] Tedesco LA, Albino JE, Cunat JJ, Green LJ, Lewis EA, Slakter MJ (1983). A dental-facial attractiveness scale: part I. Reliability and validity. Am J Orthod.

[35] Howells DJ, Shaw WC (1985). The validity and reliability of ratings of dental and facial attractiveness for epidemiologic use. Am J Orthod.

